# Supplementary material for: Comprehensive characterization of the antibody responses to SARS-CoV-2 Spike protein finds additional vaccine-induced epitopes beyond those for mild infection
Source: eLife. 2022 Jan 24;11:e73490. doi: 10.7554/eLife.73490 (PMC8887901; doi:10.7554/eLife.73490)
Supplement: Transparent reporting form [file elife-73490-transrepform1.docx]

***eLife’s* transparent reporting form**

We encourage authors to provide detailed information *within their submission* to facilitate the interpretation and replication of experiments. Authors can upload supporting documentation to indicate the use of appropriate reporting guidelines for health-related research (see [EQUATOR Network](http://www.equator-network.org/%20)), life science research (see the [BioSharing Information Resource](https://biosharing.org/)), or the [ARRIVE guidelines](http://www.plosbiology.org/article/info:doi/10.1371/journal.pbio.1000412) for reporting work involving animal research. Where applicable, authors should refer to any relevant reporting standards documents in this form.

If you have any questions, please consult our Journal Policies and/or contact us: [editorial@elifesciences.org](mailto:editorial@elifesciences.org).

**Sample-size estimation**

- You should state whether an appropriate sample size was computed when the study was being designed

N/A

- You should state the statistical method of sample size computation and any required assumptions

N/A

- If no explicit power analysis was used, you should describe how you decided what sample (replicate) size (number) to use

No explicit power analysis was used to to decide on a sample size. Samples were obtained as described in **Methods:** Sample collection. And were limited by samples available from ongoing HAARVI, and NIH Moderna trial studies. Results depended on inferential statistical tests of mean diffference. Sample size is factored into the estimates of significance and need not be otherwise justified.

Please outline where this information can be found within the submission (e.g., sections or figure legends), or explain why this information doesn’t apply to your submission:

**Replicates**

- You should report how often each experiment was performed

All sample annotation, including date of each sample IP run, can be found	at <https://github.com/matsengrp/phage-dms-vacc-analysis> . The dates of all samples	run range from 5/4/2020 - 4/7/2021

- You should include a definition of biological versus technical replication

See Manuscript:

**MATERIALS AND METHODS**

-Sample curation and replicate structure

- The data obtained should be provided and sufficient information should be provided to indicate the number of independent biological and/or technical replicates

See Manuscript:

**MATERIALS AND METHODS**

-Sample curation and replicate structure

-Sample collection

- If you encountered any outliers, you should describe how these were handled

N/A

- Criteria for exclusion/inclusion of data should be clearly stated

See Manuscript:

**MATERIALS AND METHODS:**

Sample curation and replicate structure

- High-throughput sequence data should be uploaded before submission, with a private link for reviewers provided (these are available from both GEO and ArrayExpress)

See Manuscript:

**CODE AND DATA AVAILABILITY**

Please outline where this information can be found within the submission (e.g., sections or figure legends), or explain why this information doesn’t apply to your submission:

**Statistical reporting**

- Statistical analysis methods should be described and justified

  See Manuscript:

**STATISTICAL ANALYSIS**

- Raw data should be presented in figures whenever informative to do so (typically when N per group is less than 10)

  See Manuscript:

**RESULTS**

- For each experiment, you should identify the statistical tests used, exact values of N, definitions of center, methods of multiple test correction, and dispersion and precision measures (e.g., mean, median, SD, SEM, confidence intervals; and, for the major substantive results, a measure of effect size (e.g., Pearson's r, Cohen's d)

See Manuscript:

**RESULTS**

**STATISTICAL ANALYSIS**

- Report exact p-values wherever possible alongside the summary statistics and 95% confidence intervals. These should be reported for all key questions and not only when the p-value is less than 0.05.

See Manuscript:

**RESULTS**

**FIGURE 2**
**FIGURE 3**
**CODE AND DATA AVAILABILITY**

Please outline where this information can be found within the submission (e.g., sections or figure legends), or explain why this information doesn’t apply to your submission:

**STATISTICAL ANALYSIS**

(For large datasets, or papers with a very large number of statistical tests, you may upload a single table file with tests, Ns, etc., with reference to sections in the manuscript.)

**Group allocation**

- Indicate how samples were allocated into experimental groups (in the case of clinical studies, please specify allocation to treatment method); if randomization was used, please also state if restricted randomization was applied

  See Manuscript:

**MATERIALS AND METHODS**

Subsection - Sample curation and replicate structure

Subsection - Sample collection

**CODE AND DATA AVAILABILITY**

- Indicate if masking was used during group allocation, data collection and/or data analysis

  No masking of the data was applied. Data was normalized to a batch specifric phage library input peptide frequency as described in **MATERIALS AND METHODS**

Please outline where this information can be found within the submission (e.g., sections or figure legends), or explain why this information doesn’t apply to your submission:

**Additional data files (“source data”)**

- We encourage you to upload relevant additional data files, such as numerical data that are represented as a graph in a figure, or as a summary table
- Where provided, these should be in the most useful format, and they can be uploaded as “Source data” files linked to a main figure or table
- Include model definition files including the full list of parameters used
- Include code used for data analysis (e.g., R, MatLab)
- Avoid stating that data files are “available upon request”

Please indicate the figures or tables for which source data files have been provided:

See Manuscript:

**CODE AND DATA AVAILABILITY**

All data, code, and parameters used can be found at

<https://github.com/matsengrp/phage-dms-vacc-analysis>
